# Lipid Profile and Triglyceride-Glucose Index (TyG) Alterations in a Single-Center Cohort of Children Diagnosed with Central Precocious Puberty

**DOI:** 10.3390/children11060639

**Published:** 2024-05-25

**Authors:** Giorgio Sodero, Lucia Celeste Pane, Elena Malavolta, Giulia Rotunno, Linda Sessa, Barbara Fraccascia, Marcello Candelli, Donato Rigante, Clelia Cipolla

**Affiliations:** 1 Department of Life Sciences and Public Health, Fondazione Policlinico Universitario A. Gemelli IRCCS, 00168 Rome, Italy; giorgio.sodero01@icatt.it (G.S.); luciaceleste.pane01@icatt.it (L.C.P.); clelia.cipolla@policlinicogemelli.it (C.C.); 2 Department of Emergency Anesthesiological and Reanimation Sciences, Fondazione Policlinico Universitario A. Gemelli IRCCS, 00168 Rome, Italy; marcello.candelli@policlinicogemelli.it; 3 Università Cattolica Sacro Cuore, 00168 Rome, Italy

**Keywords:** central precocious puberty, cholesterol, triglycerides, triglyceride-glucose index, child

## Abstract

*Background:* A correlation between plasma lipids and timing of pubertal development has been hypothesized, though lipid influence remains unclear in central precocious puberty (CPP). *Aim:* To assess any possible alterations in the lipid profile and triglyceride glucose index (TyG) in children diagnosed with CPP. *Patients and Methods:* Retrospective single-center study conducted on children (aged 6.3 ± 2.1 years) evaluated for the suspicion of CPP. *Results:* Based on the results of the gonadotropin releasing hormone (GnRH) test, considering 5 IU/L as cut-off of the luteinizing hormone peak, CPP was confirmed in 43 patients (57.3%). Sixteen (37.2%) had a pathologic body mass index (BMI), with 9 (20.9%) being overweight and 7 (16.27%) obese. High total cholesterol was found in 3 patients with CPP (6.97%), high triglycerides were found in 11 patients with CPP (25.58%), high LDL cholesterol was found in 5 patients with CPP (11.62%), low HDL cholesterol was found in 12/43 patients with CPP (27.9%), a pathologic TyG was found in 13/43 patients with CPP (30.23%). No significant association was observed in the lipid profile for patients with or without CPP, except for HDL cholesterol, which was lower in the CPP group (47.1 ± 10.9; *p* = 0.033). However, the association between serum HDL cholesterol and CPP was not confirmed at the multivariate logistic regression analysis adjusted for patients’ sex and age (*p* = 0.1; OR: 1.035; 95% CI: 0.993–1.078). *Conclusion:* The overall lipid profile of our pediatric patients diagnosed with CPP did not differ from patients having idiopathic precocious thelarche or normal variants of puberty development.

## 1. Introduction

Central precocious puberty (CPP) has been traditionally defined as the onset of secondary sexual characteristics before 8 years in males or before 9 years in females [1]. In recent years there have been increased reports of CPP at the same time of severe acute respiratory syndrome coronavirus pandemic [2], with a considerable rise in outpatient visits for this particular suspicion, and concurrent increased diagnosis of CPP through the gonadotropin releasing hormone (GnRH) test [3]. Children with suspected CPP are initially subjected to thorough auxological evaluation for monitoring growth velocity (which is typically increased), height percentile (consistent with early pubertal growth spurt), and onset or progression of the secondary sexual characteristics, with breast development (thelarche) generally being the first sign in females and testicular enlargement in males [4]. Additional tests encompass baseline measurements of gonadotropins, sex hormones, and a general screening that includes thyroid function to distinguish true CPP cases from thelarche and normal variants of pubertal development [1]. Other complementary assessments include evaluation of bone age (which results typically advanced in comparison with the chronological age) and pelvic ultrasound, which reveals increased size and volume of the genital tract in some cases [5].

In recent years there have been studies investigating a potential correlation between body mass index (BMI) and timing of pubertal development, as cytokines and hormones activated in obese patients appear to influence the hypothalamic-pituitary-gonadal axis, leading to early achievement of full-blown puberty [6]. Several publications have highlighted how overweight and obesity in childhood may act as triggers for CPP [7]. Furthermore, BMI has been statistically correlated with the peak of luteinizing hormone (LH) [1]. Subsequently, alterations in plasma lipids due to unhealthy dietary habits, typical of overweight patients, might bring about systemic low-grade chronic inflammation, hypothalamic activation, and subsequent inception of CPP [8].

The exact role of cholesterol and triglycerides in patients with CPP is yet unclear, and a general screening of plasma lipids is usually performed before GnRH test, as early puberty correlates with overweight, increasing the cardiovascular risks of such patients [9]. However, there is limited information about CPP in the presence of a dysregulated lipid metabolism both in male and female patients. Furthermore, it should be considered that diagnosis of CPP, while being standardized by international guidelines, is made differently based on both experience of pediatric endocrinologists and specific centers that manage patients with endocrinopathies. For example, the GnRH test, despite being considered the diagnostic gold standard, may be interpreted with different cut-off values across several nations. Indeed, there are significant differences in the execution of the test itself, with some recent studies showing that simplified methods present a diagnostic accuracy similar to the classic one [10]. Consequently, the interpretation and identification of new parameters that could guide and raise the suspicion of CPP are crucial. Additionally, given the increased cardiovascular risk associated with early pubertal activation, it is also fundamental to modify all parameters that may contribute to further morbidity for these patients [11,12].

Based on these starting considerations, we performed a single-center study aimed at analyzing any potential alterations of the lipid profile and triglyceride glucose index (TyG) in children assessed for CPP.

## 2. Patients and Methods

This retrospective study was conducted between January 2022 and April 2024 on patients evaluated at the Pediatric Endocrinology Day Hospital of the Fondazione Policlinico Universitario A. Gemelli IRCCS, Rome, for the suspicion of CPP. The main objective of our study was to evaluate the presence of alterations in the general lipid profile combined with TyG among patients with a true CPP confirmed by the GnRH test (with LH peak >5 IU/L) and those with a suspected CPP but having a normal result at the GnRH test. GnRH tests included both LH and follicle stimulating hormone (FSH) measurements.

### 2.1. Ethical Approval

Ethics committee approval was not obtained because the General Authorization to Process Personal Data for Scientific Research Purposes (Authorization No. 9/2014) states that retrospective archival studies using codes that prevent direct data tracing of subjects do not require an official ethics approval. Nevertheless, all analyses and measurements performed on our patients adhered to the standards of good clinical practice and routine management of patients with suspected CPP. All parents of our patients were informed about the aims of this study, and after being recalled they also provided a written informed consent to participate.

### 2.2. Inclusion and Exclusion Criteria

We considered these inclusion criteria: age <8 years for females and <9 years for males; girls with ‘early puberty’ (defined as *development of secondary sexual characteristics between 8 and 9 years*) were excluded from our study analysis, as *early puberty* is considered a distinct condition from CPP, being interpreted as a normal variant of physiologic puberty [12]. The suspicion of CPP was confirmed by the pediatric endocrinology assessment (premature thelarche with or without pubarche, advanced bone age, pubertal findings on the pelvic ultrasound assessment, height and/or growth velocity above the upper limits for patient’s age based on the national reference charts).

Patients’ follow-up at our center included auxological parameters (weight, height, and BMI; for each variable we calculated the respective standard deviation [SD] for age and sex to make measurements comparable among children of different age), laboratory tests consisting of complete thyroid profile [thyroid-stimulating hormone (TSH), triiodothyronine (fT3), and thyroxine (fT4)], glucose, lipid profile including total cholesterol, triglycerides, low-density lipoprotein (LDL) cholesterol, high-density lipoprotein (HDL) cholesterol and TyG, and radiological data (i.e., brain imaging; ovarian and uterine diameters/volumes were also evaluated by transabdominal pelvic ultrasound in females). All blood samples were collected after overnight fast and prior to start treatment for CPP.

This screening process enabled us to select 75 consecutive patients with a suspected CPP: the mean age of all selected children was 6.2 ± 2.0 years. A true CPP was confirmed by the GnRH test if the LH peak exceeded 5 IU/L.

### 2.3. References for Anthropometric and Biochemical Parameters

According to the diagnostic criteria of the World Health Organization [13,14] and BMI categories approved by the Centers for Disease Control and Prevention (overweight if BMI percentile is ≥85th and <95th; obesity if BMI percentile is ≥95th percentile) [15,16], among our patients with CPP, 16 (37.2%) had an increased BMI, with 9 (20.9%) being overweight and 7 (16.3%) obese; among patients without CPP, 16 (47.1%) had an increased BMI, with 12 (35.3%) being overweight and 4 (11.8%) obese. None of the patients in both groups exhibited a reduced BMI. According to the Italian Society of Diabetology [17] we considered fasting glucose ≤ 100 mg/dL as a normal value; none of our patients had pathological fasting glucose results.

According to the Expert Panel on Integrated Guidelines for Cardiovascular Health and Risk Reduction in Children and Adolescents [18], we divided plasma lipid and lipoprotein concentrations (mg/dL) into the following categories: total cholesterol acceptable if <170, borderline if between 170–199, high if ≥200; LDL cholesterol acceptable if <110, borderline if between 110–129, high if ≥130; HDL cholesterol acceptable if >45, borderline if between 40–45, low if <40; triglycerides acceptable if <75, borderline if between 75–99, high if >100.

### 2.4. Sample Size Assessments

Our sample was assessed considering the differences in total cholesterol levels between patients with or without CPP, considering a mean value of 160 mg/dL with a standard deviation of 12 in normal subjects and expecting a mean of at least 10 mg/dL higher among patients with CPP, with an alpha error of 0.05, a beta error of 0.2, and therefore a power of 80%. The minimum sample size required for each group was 23 subjects.

### 2.5. Statistical Analysis

To perform the statistical analysis we used the IBM SPSS software version 25. Continuous variables were described using the mean and standard deviation. Comparisons between continuous variables were conducted using the Student’s *t*-test (for normally distributed data). Categorical variables were described using frequency and percentage of the total. In this case, we made group comparison using the chi-square test or Fisher’s exact test, as appropriate, based on the group sizes. To adjust our comparisons for confounding factors we conducted a multivariate logistic regression analysis using as covariates HDL cholesterol, BMI, TyG, and triglycerides and correcting for patients’ sex and age. A *p* value < 0.05 was considered statistically significant.

## 3. Results

We analyzed data from 75 patients (3 males and 72 females). The mean age was 6.26 ± 2.06 years. Table 1 resumes the auxological and laboratory characteristics of our complete cohort of patients.

Based on the results of the GnRH test, considering a cut-off of 5 IU/L for the LH peak, CPP was confirmed in 43 patients (57.3%); a diagnosis of CPP was excluded in 32 patients (42.7%), who were considered to have isolated early thelarche or non-pathological variants of pubertal development. Diagnosis of CPP was established in 1 male patient and in 42/72 females (58.3%). Brain magnetic resonance imaging (MRI) was performed to exclude an underlying etiology in all 43 cases of CPP. In 39 out of 43 cases (90.7%) CPP was considered ‘idiopathic’, while an underlying organic pathology was found in 4/43 patients (9.3%), i.e., central nervous system tumors in the form of noninvasive low-grade gliomas. No patient had comorbidities related to overweight or obesity (such as cardiovascular, hepatic, or renal diseases). Treatment with GnRH analogs was prescribed in 100% of patients with a confirmed CPP, following our local guidelines [12].

At the endocrinological follow-up, six months later, a favorable response to therapy was observed in all cases with regression of secondary sexual characteristics and/or cessation of pubertal development. The families of all patients did not report any significant side effects or complications.

**Figure 1:**
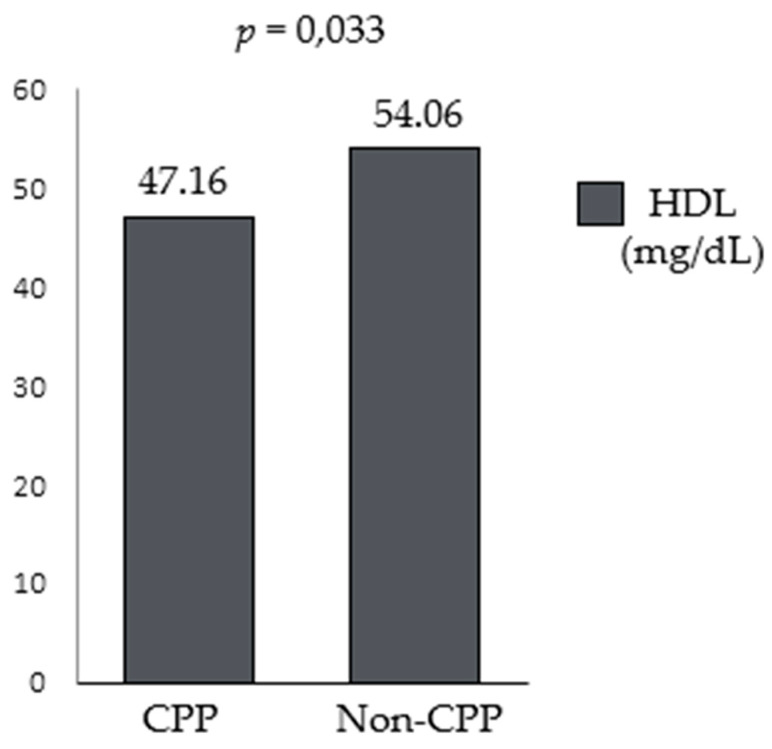
Difference in mean high density lipoprotein (HDL) cholesterol levels between patients with and without central precocious puberty (CPP).

We subsequently classified patients upon the results of anthropometric and previously described labwork cut-offs, obtaining these results: high total cholesterol was found in 3/43 patients with CPP (6.97%) and in 2/32 patients without CPP (6.25%); high LDL cholesterol was found in 5/43 patients with CPP (11.62%) and in 1/32 patients without CPP (3.12%); lower HDL cholesterol was found in 12/43 patients with CPP (27.90%) and in 7/32 without CPP (21.87%); high triglycerides were found in 11/43 patients with CPP (25.58%) and in 4/32 patients without CPP (12.5%). A pathological TyG was found in 13/43 patients with CPP (30.23%) and in 5/32 without CPP (15.62%). All patients with a pathological concentration of lipids were females. Based on our classification criteria, we also analyzed the potentially significant differences between the two groups. The Chi-Square Test was performed to see whether there was any association between pathologic BMI *z*-scores (overweight or obese), high total cholesterol, high LDL cholesterol, low HDL cholesterol, high triglycerides, high TyG and CPP. No significant association was found between pathologic BMI (*p* = 0.22), high total cholesterol (*p* = 1.00), high LDL cholesterol (*p* = 0.39), high triglycerides (*p* = 0.24), high TyG (*p* = 0.16), low HDL cholesterol (*p* = 0.62) and CPP. In addition, T-test was performed to assess whether there was a difference in the means of BMI *z*-scores, total cholesterol, LDL cholesterol, HDL cholesterol, triglycerides, and TyG between CPP and non-CPP patients. No statistically significant differences were observed between the two groups of children, with the exception of HDL cholesterol, (47.16 ± 10.98 in the CPP group, 54.06 ± 16.46 in the non-CPP group, *p* = 0.033; Figure 1).

Considering all our patients with diagnosis of CPP, we subsequently performed a multivariate logistic regression to test the association between HDL cholesterol and CPP after adjusting for sex and age the following factors: BMI, HDL cholesterol, triglycerides, and TyG as potential independent variables. However, none of these factors were found to be associated with CPP (Table 2).

In addition to these first results, we conducted a subgroup analysis excluding male patients: considering the same parameters previously mentioned, no statistically significant differences were observed between the two subgroups of females with or without CPP at the univariate analysis (see Table 3).

In the same subgroup, a multivariate linear logistic regression analysis was performed including variables that had a *p* value <0.15 at the univariate analysis after adjustment for age. The BMI was found to be significantly correlated with CPP (see Table 4).

## 4. Discussion

CPP is mostly occurring in girls, usually resulting idiopathic; in boys CPP is a frequent manifestation secondary to neoplastic diseases [1]. Early pubertal development may work as a risk factor for the future development of both metabolic syndrome and metabolic dysfunction-associated steatotic fatty liver disease in adulthood [19]. Furthermore, the pubertal progression with onset of menarche before a physiological timing magnifies a future cardiovascular risk in these patients, who even might display higher rates of myocardial infarction [20,21].

Our retrospective single-center study was performed to assess whether pediatric patients diagnosed with CPP had any associated plasma lipid abnormalities. Cholesterol and triglyceride metabolites participate in manifold physiological processes and have been shown to affect both development and evolution of protean diseases characterized by systemic severity: for instance, lipid abnormalities have been found to regulate polarization and inflammatory activation of macrophages [22]. The search for novel mediators associated with premature signs of puberty should be pivotal for timing of endocrinological stimulation tests [12,23]. To date, CPP is diagnosed through a multi-disciplinary approach based on clinical, radiological, and hormonal data [1,12]. The relationship between obesity and CPP is well-established and confirmed by several past and more recent studies: it is commonly known that obese children exhibit an earlier onset of puberty compared to normal-weight ones [24,25,26], regardless of the confirmation of CPP diagnosis, and this association remains true for obese patients as well as for those who are overweight or at risk to develop metabolic syndrome. The mechanisms involved in such process are not fully understood, although the role of leptin in stimulating GnRH secretion by hypothalamus has been suggested [6]. On the other hand, early pubertal development increases the risk of obesity and development of diabetes mellitus type 2, while it is unclear whether pharmacological treatment with GnRH analogs could at least normalize this risk [20]. Indeed, therapy with GnRH analogs might, in some cases, worsen the metabolic profile, causing a short-term increase of plasma triglycerides and LDL cholesterol, and also worsening body fat distribution [25].

Despite the known association with obesity, confirmed also in our statistical analysis, less is deciphered about the relationship between lipid metabolism and CPP. High-fat diet might potentially induce early pubertal development via indirect activation of the hypothalamic-pituitary axis, different alterations in the intestinal microbiota, and multiple hormone dysregulation [26]. Premature adrenarche, among all signs of pubertal activation, is the most significantly correlated with a cardiovascular risk [27], as adrenal activation can lead to long-term insulin resistance [28]. However, adrenarche identifies adrenal axis activation and remains a marginal sign of CPP, while the cardiovascular risk of patients with early thelarche is less defined.

A recent study analyzed the eventual differences between patients with LH peak >5 IU/L during GnRH and patients with LH below this cut-off, highlighting how there were no significant differences between the two groups for total cholesterol, LDL cholesterol, and HDL cholesterol. However, the same authors pointed out that triglycerides were lower in the group of girls diagnosed with CPP (85.6 ± 42.7 mg/dL *versus* 112.2 ± 72.0 mg/dL, *p* = 0.023). As a collateral finding, they also highlighted that sex hormone binding globulin (SHBG), a glycoprotein that binds to sex hormones, was positively correlated with HDL cholesterol, and that this association could be justified by the fact that low circulating SHBG levels are correlated with body fat mass and hyperaccumulation of abdominal fat [29].

Sørensen et al. conducted a study similar to ours, comparing 23 girls with early puberty (of whom 13 had a subsequent diagnosis of CPP) with a control group of 115 patients, highlighting that patients with CPP had an adverse metabolic status with high triglycerides and high LDL cholesterol at the time of diagnosis [30]. Our statistical analysis did not reveal this correlation, but our sample included a higher number of patients with CPP (46 *versus* 13), in whom lower HDL levels were found.

A recent analysis conducted on pediatric patients has demonstrated that HDL levels were inversely correlated with patients’ BMI, while a direct correlation could be found with other lipid indices [31]. These observations are consistent with the general characteristics of patients with CPP, who typically tend to have higher BMI compared to children with normal pubertal development [1,4]. However, our statistical analysis did not reveal correlations with lipid indices, though a metabolic dysregulation could generate a systemic inflammatory status capable of prematurely activating the hypothalamic-pituitary axis in both overweight and obese patients [25]. It is known that molecules such as insulin can directly or indirectly act via insulin growth factor-1 on the central nervous system [6]: this activation also affects adipocytes, which are stimulated to proliferate and produce adiponectins, contributing to perpetuating the inflammatory process characterized by recruitment of immune and non-immune cells in CPP [26].

Despite several articles regarding the correlation between lipid profile and CPP, there are few papers correlating indirect indices, such as TyG, an index of insulin resistance, with results of the GnRH test [32]. Alterations in carbohydrate metabolism eventually associated with obesity may also influence early puberty and are not improved by therapy with GnRH analogs [33]. Unfortunately, our statistical analysis did not reveal significant differences for this index. Jiang et al. conducted a systematic review with meta-analysis on papers evaluating lipid profile in patients with CPP [34]. Considering a total of 14 studies involving 1023 girls with pathological GnRH tests, triglycerides, total cholesterol, and LDL cholesterol levels were found to be significantly elevated in girls with CPP (*p* = 0.04, *p* = 0.04, *p* = 0.02, respectively), while HDL cholesterol did not change significantly (*p* = 0.62). Although no differences were observed, the lipid profile might change following treatment with GnRH analogues, as these drugs can cause hyperlipidemia in the short term [25].

The search for parameters useful in differentiating patients with CPP from those with normal variants of pubertal development is important as clues for diagnosis of early activation of the hypothalamic-pituitary-gonadal axis are not yet standardized to date [1,35], and any potential differences could be helpful to exactly identify cases needing medical assessments with a GnRH test. Our statistical analysis showed no significant differences in the lipid profile between children having CPP and not: in fact, despite HDL cholesterol was lower in the CPP group, this association was not confirmed at the multivariate analysis after adjustment for confounding factors.

### Strength and Limitations of the Present Study

The main strength of our study is the consecutive inclusion of all patients with a suspected CPP, ensuring representativeness and preventing selection biases. Additionally, the number of patients is notable compared to other similar studies in the medical literature.

Limitations of this study are as follow: (a) it is a retrospective monocentric study, and its results may not be generalizable to the general population; (b) our sample, in line with the incidence of CPP, consists almost exclusively of females (therefore, future studies conducted on a larger number of children with idiopathic CPP may better characterize the lipid profile in male patients); (c) the lack of a statistical correlation between the overall lipid profile and CPP allowed us to exclude alterations in our sample. Additionally, we did not perform a subgroup analysis considering patients’ ethnicity, as pubertal development may be also influenced by the geographical origin [36,37]. In our cohort the majority of patients were Caucasian; therefore, it was not possible to conclude this secondary analysis. The study conducted by Jiang et al. [34] revealed that triglycerides were higher in European girls with CPP compared to controls, but did not differ in the subgroups of American and Asian girls; furthermore, these authors noted that triglyceride levels did not correlate with CPP in all subgroups.

## 5. Conclusions

In our sample of pediatric patients diagnosed with CPP the overall lipid profile did not differ from those with idiopathic precocious thelarche and normal variants of puberty. Future investigations could probably focus on further laboratory data of individuals with CPP, emphasizing any variations in both metabolic and hormonal profile. This approach should enhance the comprehension regarding the influence of lipid abnormalities in children with CPP and also reduce their cardiovascular risk in light of early pubertal activation.

## Figures and Tables

**Table 1:** Auxological and laboratory characteristics of the cohort of patients with central precocious puberty.

	Mean ± SD
Number of children (N)	75
Age (years)	6.3 ± 2.1
Basal LH (IU/L)	0.9 ± 1.36
Basal FSH (IU/L)	4.14 ± 3.19
LH peak (IU/L)	10.50 ± 12.57
FSH peak (IU/L)	17.11 ± 14.60
Peak LH/FSH ratio	0.58 ± 0.69
BMI *z*-score	0.79 ± 1.49
Glucose (mg/dL)	75 ± 8
Total cholesterol (mg/dL)	156 ± 26
HDL cholesterol (mg/dL)	50 ± 14
LDL cholesterol (mg/dL)	91 ± 24
Triglyceride glucose index (TyG)	4.27 ± 0.22
TSH (microIU/mL)	3.14 ± 1.84

*Legend:* N number; LH luteinizing hormone; FSH follicle-stimulating hormone; BMI body mass index; HDL high density lipoprotein; LDL low density lipoprotein; TSH thyroid stimulating hormone.

**Table 2:** Multivariate logistic regression analysis to examine the association between high density lipoprotein (HDL) cholesterol levels and central precocious puberty after adjusting for sex and age, including body mass index (BMI), HDL cholesterol, triglycerides, and triglyceride-glucose index (TyG).

Variable	*p*	OR	95% CI
Age	0.335	0.875	0.666–1.148
Sex (M)	0.709	1.640	0.122–22.020
BMI	0.091	1.138	0.980–1.322
HDL cholesterol	0.101	1.035	0.993–1.078
Triglycerides	0.708	0.992	0.949–1.036
TyG	0.848	1.905	0.003–14.051

*Legend:* OR odds ratio; CI confidence interval; M male; HDL high density lipoprotein.

**Table 3:** Lipid profile in female patients with and without central precocious puberty (CPP).

Variable	CPP Females	Non-CPP Females	*p* Value
Total cholesterol	155.88 ± 26.6	155.20 ± 26.2	0.67
LDL cholesterol	94.52 ± 24.8	87.27 ± 20.8	0.18
HDL cholesterol	47.12 ± 11.1	53.23 ±16.3	0.08
Triglycerides	83.00 ±39.9	69.03 ± 31.2	0.1
TyG	4.31 ± 0.2	4.24 ± 0.2	0.2
BMI	16.70 ± 2.6	18.86 ± 7.0	0.12

*Legend:* BMI body mass index; HDL high density lipoprotein; LDL low density lipoprotein; TyG triglicerides glucose index.

**Table 4:** Multivariate linear logistic regression to assess any correlation between the lipid profile and central precocious puberty in female patients.

Variable	*p* Value
BMI	0.048
HDL cholesterol	0.109
Triglycerides	0.619
Age	0.373

*Legend:* BMI body mass index; HDL high density lipoprotein.

## Data Availability

No new data were created or analyzed in this study; data sharing is not applicable to this article.
